# *Dianthus aticii*, a new species from Turkey (Caryophyllaceae)

**DOI:** 10.3897/phytokeys.48.4446

**Published:** 2015-04-02

**Authors:** Ergin Hamzaoğlu, Murat Koç, Ahmet Aksoy

**Affiliations:** 1 Department of Elementary Education, Gazi Faculty of Education, Gazi University, 06500, Ankara, Turkey; 2 Animal Production High School, Bozok University, 66900, Yozgat, Turkey; 3 Department of Biology, Science Faculty, Akdeniz University, 07058, Antalya, Turkey

**Keywords:** *Dianthus*, new species, section *Dentati*, taxonomy, Turkey

## Abstract

During the taxonomic revision of the Turkish *Dianthus* species, specimens collected from Bilecik, Seben (Bolu), and Nallıhan (Ankara) were discovered that represent a new species. Its description, images, chorology, ecology, and threat category are provided. It was compared with a closely related species, *Dianthus zonatus*, and differences are based on its general morphology and seed micromorphology.

## Introduction

The genus *Dianthus* L. (or carnation, pink, sweet william) is easily distinguished from the other genera in the family Caryophyllaceae by its epicalyx scales, tubular calyx, and peltate seeds. Furthermore, it is the second largest genus of the family in Turkey, with the highest number of species after *Silene* L. (Reeve 1967, Bojňanský and Fargašová 2007). This genus consists of approximately 300 species and is mainly distributed across the Mediterranean region of Europe and Asia (Bittrich 1993).

The most comprehensive taxonomic revision of *Dianthus* species of Turkey was carried out by Reeve (1967), who recognized 67 species from this region. Since Reeve’s revision, nine species have been added (Davis et al. 1988, Gemici and Leblebici 1995, Güner 2000, Menemen and Hamzaoğlu 2000, Aytaç and Duman 2004, Özhatay and Kültür 2006, Vural 2008, Yılmaz et al. 2011, İlçim et al. 2013), bringing the current total to 76 species.

In the course of performing a taxonomic revision of *Dianthus* species of Turkey, specimens from Bilecik, Seben (Bolu), and Nallıhan (Ankara) were identified as *Dianthus zonatus* Fenzl based on their appearance and present-day key characteristics. However, on further study, it was revealed that they belonged to a new species. This study was undertaken to recognize this new species and to characterize the differences between these two species.

The specimens collected from Bilecik, Seben (Bolu), and Nallıhan (Ankara) were found to be distinct from those of *Dianthus zonatus* on the basis of vegetative, floral, and seed characteristics (Table 1). Vegetative characters of the new species include a suffruticose habit as opposed to herbaceous in *Dianthus zonatus*, and leaves that are subulate and subcanaliculate rather than linear and flattened. Floral characters of the epicalyx and petals are smaller than those of *Dianthus zonatus*, and seed shape and seed cell traits are distinctive between the two species. Based on these differences, it was clear that these specimens represent a previously undescribed species.

**Table 1.** Diagnostic characters between *Dianthus aticii* and *Dianthus zonatus*.

Characters	*Dianthus aticii*	*Dianthus zonatus*
Stems	suffruticose	herbaceous
Sterile shoot leaves	subulate, subcanaliculate	linear, flattened
Cauline leaves	subcanaliculate, subulate to linear-filiform	flattened, linear to linear-filiform
Outer epicalyx scales	4–8 mm long	5–15 mm long
Inner epicalyx scales	6–9 mm long, scarious margin 0.2–0.4 mm wide, arista 1/10–1/7 as long as scale	8–16 mm long, scarious margin 0.4–0.8 mm wide, arista 1/6–1/3 as long as scale
Petals	20–23 mm long; limbs 7–8 mm long; claw 12–15 mm long	24–31 mm long; limbs 8–10 mm long; claw 16–21 mm long
Seed shape	elliptical	suborbicular
Cell edges of dorsal surface of seed	V-undulate	S-undulate
Cell edges of ventral surface of seed	S-undulate	V-undulate

## Materials and methods

*Dianthus* specimens were thoroughly evaluated using the relevant literature (Fenzl 1842, Boissier 1849, Tchihatcheff 1860, Reeve 1967, Bojňanský and Fargašová 2007) and the specimens present in GAZI herbarium. Furthermore, the specimens collected from the Bilecik population included the material needed for the seed micromorphology studies. Images were taken using a Canon EOS 60D digital camera, and the seed surface micromorphology was visualized using a LEO 440 scanning electron microscope. Normal visualization of the specimens was carried out using a Leica EZ4 HD microscope. The vegetative characters were measured using a ruler with 0.5 mm accuracy; floral characters were measured using an ocular micrometer. Seed morphology is described following the nomenclature of Bojňanský and Fargašová (2007). Specimens have been deposited in the herbaria of Gazi (GAZI) and Ankara Universities (ANK).

## Taxonomic treatment

### Dianthus aticii

Taxon classificationPlantaeCaryophyllalesCaryophyllaceae

Hamzaoğlu sp. nov.

urn:lsid:ipni.org:names:77146130-1

Figs 1 , 2

#### Diagnosis.

Stems suffruticose (not herbaceous); sterile shoot leaves subulate, subcanaliculate (not linear, flattened or absent); inner epicalyx scales with scarious margin 0.2–0.4 mm wide, arista 1/10–1/7 as long as scale (not with scarious margin 0.3–0.8 mm wide, arista 1/7–1/3 as long as scale).

#### Type.

**TURKEY. Bilecik:** Bilecik highway exit towards Eskişehir, 40°06'27"N, 29°59'47'E, 330 m, stony slopes and steppes, 16 June 2013 (fl, fr), *E. Hamzaoğlu et al. 6743* (holotype: GAZI; isotypes: GAZI, ANK); **Bolu:** Seben, between Bozyer and Korucuk villages, 1025 m, forest clearings, flowing slopes, 19 July 2013, *M. Koç & E. Hamzaoğlu 6868* (paratypes: GAZI, ANK); **Ankara:** Nallıhan, Gökçeöz village, road of forest watchtower, 820 m, forest clearings, stony slopes, 19 July 2013, *M. Koç & E. Hamzaoğlu 6869* (paratypes: GAZI, ANK).

#### Specimens examined.

***Dianthus aticii* Hamzaoğlu sp. nov.** – **TURKEY. Bilecik:** Bilecik highway exit towards Eskişehir, 40°06'27"N, 29°59'47'E, 330 m, stony slopes and steppes, 16 June 2013, *E. Hamzaoğlu et al. 6743* (holotype: GAZI; isotypes: GAZI, ANK); **Bolu:** Seben, between Bozyer and Korucuk villages, 1025 m, forest clearings, flowing slopes, 19 July 2013, *M. Koç & E. Hamzaoğlu 6868* (paratypes: GAZI, ANK); **Ankara:** Nallıhan, Gökçeöz village, road of forest watchtower, 820 m, forest clearings, stony slopes, 19 July 2013, *M. Koç & E. Hamzaoğlu 6869* (paratypes: GAZI, ANK); ***Dianthus zonatus* Fenzl** – **TURKEY. Manisa:** Spil Dağı National Park, road of Atalanı resting area, 1320 m, calcerous rocks, 2 July 2011, *M. Koç & E. Hamzaoğlu 6106* (GAZI); **Kütahya:** İscehisar, around Seydiler, 1150 m, rocks, 5 August 2012, *E. Hamzaoğlu et al. 6584* (GAZI); **Eskişehir:** Around Sivrihisar, 1115 m, rocks, 24 June 2012, *E. Hamzaoğlu et al. 6339* (GAZI); **Konya:** Between Kulu and Cihanbeyli, Kulu exit, 1130 m, steppe, 13 July 2011, *E. Hamzaoğlu et al. 6122* (GAZI); **Ankara:** Polatlı, above Babayokuş village, 900 m, stony places, 2 July 2010, *M. Koç et al. 1205* (GAZI); **Aydın:** Between Söke and Didim, after 4 km from Güllübahçe exit, 820 m, 25 June 2006, *E. Hamzaoğlu et al. 4071* (GAZI); **Muğla:** Köyceğiz, above Yayla village, from Gökçeova Lake to Sandras Mountain summit, 1950 m, serpentine rocks, 15 July 2011, *E. Hamzaoğlu et al. 6198* (GAZI); **Antalya:** Elmalı, N of Vahhabi Ümmi Türbesi, 1480 m, rocks, 12 June 2007, *M. Koç & Ü. Budak 2152* (GAZI); **Karaman:** Between Ermenek and Karaman, 16 km, 1670 m, Pine forest openings, stony places, 18 July 2005, *Ü. Budak et al. 1743* (GAZI); **Niğde:** Çamardı, above Demirkazık village, 1475 m, rocks, 11.7.2012, *E. Hamzaoğlu et al. 6449* (GAZI).

#### Description.

Suffruticose, several-stemmed, subpruinose herbs. Stems erect, fragile, 20–35 cm tall, branching from upper nodes, 6–10-nodes, glabrous or puberulent. Leaves subcanaliculate, thick, glabrous or puberulent, margins scabrous, ciliate and scarious at base, apex acuminate; sterile shoot leaves subulate, equal or longer than cauline leaves; cauline leaves subulate to linear-filiform, 11–22 × 0.6–1.2 mm, appressed to stem, obviously shorter than internodes, rigid, 3-veined, sheaths equal or slightly longer than wide; upper similar but smaller. Flowers solitary or few in racemes; branches angled at 5–15°, glabrous or sparsely puberulent, up to 3 cm long; pedicels 5–15 mm, glabrous or sparsely puberulent, greenish. Epicalyx scales (4-)6–8(-12), cartilaginous, greenish or straw-coloured, glabrous or puberulent, appressed to calyx, apex acute to acuminate except arista; outer linear-lanceolate, veinless below, indistinctly 5–9-veined above, 1/5–2/5 as long as calyx, 4–8 × 0.8–1.2 mm, with narrowly scarious (c. 0.2 mm) margins, arista 1/2–2/3 as long as scale; inner oblong-oblanceolate, veinless below, indistinctly 7–9-veined above, 2/5–1/2 as long as calyx, 6–9 × 2.5–3.5 mm, with scarious (0.2–0.4 mm) margins, arista 1/10–1/7 as long as scale. Calyx cylindric-lanceolate, 16–22 × 3–4.5 mm, distinctly 36–40-veined above, glabrous or puberulent, pale green or sometimes purplish; teeth triangular-lanceolate, 4–5.5 × 1.2–2 mm, 7-veined, with ciliate and scarious margins, apex acute to acuminate, sometimes short mucronate. Petals 20–23 mm long; limb broadly cuneate, 7–8 × 6–7 mm, c. 1/3 as long as petal, completely exserted from calyx, usually spotted, barbulate, pink, yellowish-green beneath, 7–11-toothed to apex, teeth triangular, up to 1/6 as long as limb; claw 12–15 × 1.5 mm, collar almost as wide as claw. Capsule equal in length to calyx. Seeds elliptical, 2–3 × 1.4–2 mm, blackish.

#### Distinction from other taxa.

*Dianthus aticii* shows close similarities to *Dianthus zonatus* Fenzl because of toothed and barbulate petals, solitary or double flowers, and epicalyx scales that reach up to half of its calyx length (Fenzl 1842, Boissier 1849, Tchihatcheff 1860, Reeve 1967). Despite these similarities, there are very distinctive differences between *Dianthus aticii* and *Dianthus zonatus* such as stem morphology, leaf shape, and size of epicalyx scales and petals (Table 1, Figure 2).

#### Key to the two closely related *Dianthus* species

**Table:** 

1	Stems suffruticose; sterile shoot leaves subulate and subcanaliculate; inner epicalyx scales with scarious margin 0.2–0.4 mm wide; petals 20–23 mm long	***Dianthus aticii* sp. nov.**
–	Stems herbaceous; sterile shoot leaves linear and flattened or absent; inner epicalyx scales with scarious margin 0.3–0.8 mm wide; petals 24–31 mm long	***Dianthus zonatus***

#### Seed morphology.

Seeds of *Dianthus aticii* are elliptical, 2–3 × 1.4–2 mm, black, granular; dorsal surface convex, with regular rectangular cells, tuberculate, with 4–7 teeth on each margin, teeth V-undulate, apparent; ventral surface flat, with irregular rectangular cells, tuberculate, with 4–7 teeth on each margin, teeth S-undulate, not apparent; apex beaked. The seeds of *Dianthus aticii* are different from the seeds of *Dianthus zonatus* in terms of shape and cell edges of both the dorsal and ventral surfaces (Table 1, Figure 2).

**Figure 1.**
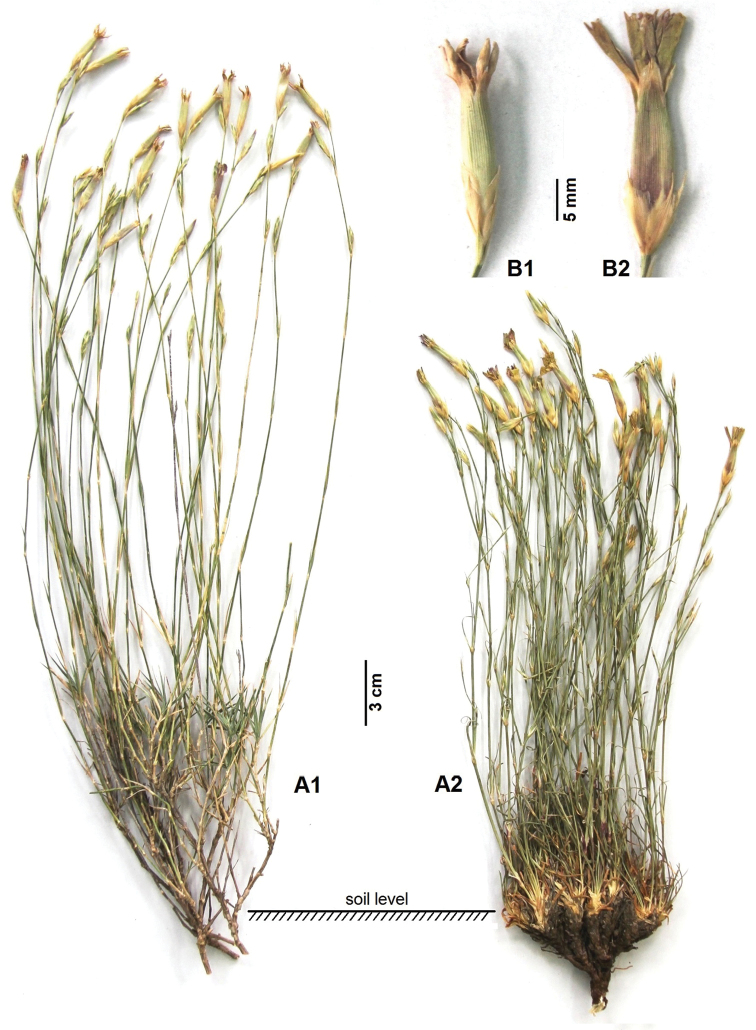
Photographs of plant habit and flowers of *Dianthus aticii* and *Dianthus zonatus*. *Dianthus aticii* – **A1** Habit **B1** Flower; *Dianthus zonatus* – **A2** Habit **B2** Flower.

**Figure 2.**
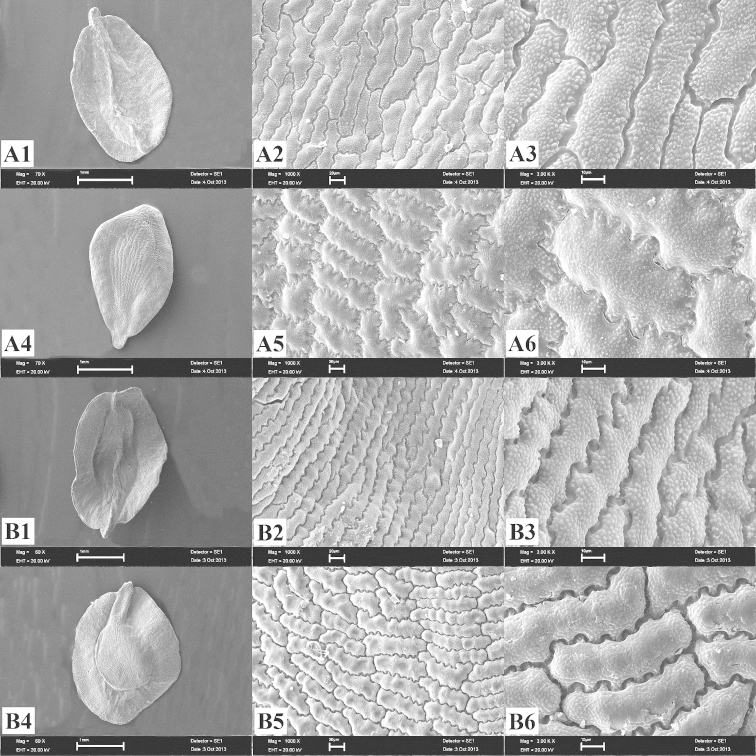
SEM photographs of the seed coat. **A** *Dianthus aticii* **B** *Dianthus zonatus* **1–3** dorsal surface **4–6** ventral surface (scale bars: **1** and **4:** 1 mm, **2** and **5:** 20 μm, **3** and **6:** 10 μm).

#### Phenology.

The new species was observed flowering in June and July, in stony slopes and steppes, between 330 and 1025 m.

#### Chorology and ecology.

*Dianthus aticii* grows in relatively sub-arid forest clearings in Bilecik, Seben (Bolu), and Nallıhan (Ankara); *it* grows in moist areas where the Euro-Siberian and Irano-Turanian phytogeographic regions coincide in the northwest part of Turkey (Davis 1965). The forest clearings of these areas that are sub-arid, compared with the oceanic climate zone, were occupied by some semi-xeric species. These areas where the forest and steppe formations co-exist are the ideal habitats for *Dianthus aticii*. The species grows on stony slopes within forest openings together with *Quercus pubescens* Willd., *Juniperus oxycedrus* L., Crataegus monogyna Jacq. subsp. monogyna, *Cistus creticus* L., *Jasminum fruticans* L., *Helianthemum nummularium* (L.) Miller, *Fumana thymifolia* (L.) Verlot, *Alyssum sibiricum* Willd., *Silene italica* (L.) Pers., *Pilosella piloselloides* (Vill.) Sojak, *Onosma tauricum* Pallas ex Willd., *Veronica multifida* L., *Teucrium polium* L., *Acantholimon acerosum* (Willd.) Boiss., *Hypericum perforatum* L., *Genista tinctoria* L., Vicia cracca L. subsp. stenophylla Vel., *Astragalus vulnerariae* DC., *Astragalus microcephalus* Willd., *Rosa canina* L., and *Centaurea urvillei* DC.

#### Conservation status.

According to the current data *Dianthus aticii* grows in the Bilecik, Seben (Bolu), and Nallıhan (Ankara) districts, which have an area of approximately 7000 km^2^. This has a discontinuous distribution due to dense forests, settlement, and farming areas. The open areas, which this species prefers, have the potential of possible settlements and agricultural activities. Therefore, the habitat of this species is under danger of being decreased and disturbed/destroyed in the future. Therefore, it is proposed that the species should be classified as *Vulnerable* [VU (B1b-iii) according to the International Union for Conservation of Nature (IUCN) categories (IUCN 2014)].

#### Etymology.

The species is named in honour of the eminent Turkish hydrobiologist Prof Dr Tahir Atıcı (Gazi Faculty of Education, Gazi University, Ankara).

## Supplementary Material

XML Treatment for Dianthus aticii
